# Trypanosomiasis

**Published:** 1908-03-21

**Authors:** 


### Tropical Diseases.

### TRYPANOSOMIASIS.

In a recent number of the "Annals of Tropical Medicine and Parasitology," Moore and Breinl, in discussing the cytology of the trypanosomes, believe they have demonstrated a latent phase in the life history of those parasites. The bodies termed by them "latent bodies" have been previously seen by different observers, but more usually have been considered forms of degeneration. The present authors, however, think that they represent a distinct phase in the life history, and further point out that instead of disappearing, they collect in the spleen and bone marrow, remaining intact there throughout the whole of the negative or latent phase of the infection when no parasites are present in the peripheral blood. It was on this assumption that mercury has been tried along with atoxyl in the treatment, the first drug being supposed to act on the latent bodies, the second on the ordinary parasites seen in the blood. This forms an interesting problem for future treatment of trypanosoma infections. Recently Plimmer has tried ordinary tartar emetic, the potassium part of the salt being replaced by sodium, in the treatment of trypanosoma infections in animals, and he believes this to be more efficacious than even atoxyl in destroying the parasites.

